# Regulation of the CCL2 Gene in Pancreatic β-Cells by IL-1β and Glucocorticoids: Role of MKP-1

**DOI:** 10.1371/journal.pone.0046986

**Published:** 2012-10-09

**Authors:** Susan J. Burke, Matthew R. Goff, Barrett L. Updegraff, Danhong Lu, Patricia L. Brown, Steven C. Minkin, John P. Biggerstaff, Ling Zhao, Michael D. Karlstad, J. Jason Collier

**Affiliations:** 1 Department of Nutrition, University of Tennessee, Knoxville, Tennessee, United States of America; 2 Sarah W. Stedman Nutrition and Metabolism Center, Duke University Medical Center, Durham, North Carolina, United States of America; 3 Advanced Microscopy and Imaging Center, University of Tennessee, Knoxville, Tennessee, United States of America; 4 Department of Surgery, Graduate School of Medicine, University of Tennessee Medical Center, Knoxville, Tennessee, United States of America; 5 University of Tennessee Obesity Research Center, Knoxville, Tennessee, United States of America; Beth Israel Deaconess Medical Center, Harvard Medical School, United States of America

## Abstract

Release of pro-inflammatory cytokines from both resident and invading leukocytes within the pancreatic islets impacts the development of Type 1 diabetes mellitus. Synthesis and secretion of the chemokine CCL2 from pancreatic β-cells in response to pro-inflammatory signaling pathways influences immune cell recruitment into the pancreatic islets. Therefore, we investigated the positive and negative regulatory components controlling expression of the CCL2 gene using isolated rat islets and INS-1-derived β-cell lines. We discovered that activation of the CCL2 gene by IL-1β required the p65 subunit of NF-κB and was dependent on genomic response elements located in the −3.6 kb region of the proximal gene promoter. CCL2 gene transcription in response to IL-1β was blocked by pharmacological inhibition of the IKKβ and p38 MAPK pathways. The IL-1β-mediated increase in CCL2 secretion was also impaired by p38 MAPK inhibition and by glucocorticoids. Moreover, multiple synthetic glucocorticoids inhibited the IL-1β-stimulated induction of the CCL2 gene. Induction of the MAP Kinase Phosphatase-1 (MKP-1) gene by glucocorticoids or by adenoviral-mediated overexpression decreased p38 MAPK phosphorylation, which diminished CCL2 gene expression, promoter activity, and release of CCL2 protein. We conclude that glucocorticoid-mediated repression of IL-1β-induced CCL2 gene transcription and protein secretion occurs in part through the upregulation of the MKP-1 gene and subsequent deactivation of the p38 MAPK. Furthermore, the anti-inflammatory actions observed with MKP-1 overexpression were obtained without suppressing glucose-stimulated insulin secretion. Thus, MKP-1 is a possible target for anti-inflammatory therapeutic intervention with preservation of β-cell function.

## Introduction

Type 1 diabetes mellitus (T1DM) results from selective elimination of the insulin-producing β-cells within the pancreatic islets via an autoimmune mediated process that requires infiltration of T-lymphocytes and activation of resident macrophages [1], [2], [3]. Accumulation of immune cells within pancreatic islets is also a major contributor to tissue rejection after islet transplantation [4], [5]. One of the primary signals leading to immune cell infiltration into tissues, including the pancreatic islets, is the release of chemotactic cytokines, usually referred to as chemokines [6], [7]. Synthesis and secretion of chemokines from the β-cell population is a major signal for islet immune cell invasion [8], [9], [10] and chemokines are critical factors associated with the development of autoimmune diabetes [11], [12], [13], [14].

One chemokine that participates in islet immune cell recruitment is CCL2, also known as monocyte chemoattractant protein-1 [15]. CCL2 is a member of the CC chemokine family and recruits specific leukocytes, such as dendritic cells, monocytes, macrophages, and T-cells to tissues from which it was initially released [13], [16]. Each of the immune cell types recruited by CCL2 influences the islet destruction that precedes onset of T1DM [17]. Polymorphisms that increase the expression of the CCL2 gene negatively correlate with pancreatic islet function [18] and transgenic overexpression of CCL2 specifically in islet β-cells promotes insulitis and progression to diabetes in the B6D2 genetic background [8]. Alternatively, transgenic expression of CCL2 in the NOD mouse decreases autoimmune-mediated β-cell destruction [19]. Thus, recruitment of leukocytes into the islet can lead to either immune cell-mediated destruction of the pancreatic β-cell or sparing of β-cell mass through non-destructive insulitis, depending on the genetic environment. Hence, understanding the molecular determinants controlling expression of the CCL2gene may offer insights into the factors regulating islet immune cell invasion.

One of the major signals controlling expression of the CCL2 gene is the cytokine IL-1β.The pro-inflammatory outcomes associated with IL-1β are often signaled through the NF-κB pathway [20]. NF-κB is composed of dimers of the transcriptional regulatory subunits RelA/p65, RelB, c-Rel, p50, and p52. The inhibitor of κB proteins (IκBs) bind to NF-κB proteins and mask their nuclear localization signal which promotes cytosolic retention [21]. Upon activation of a cell surface receptor, such as the IL-1R, a variety of signaling pathways are activated, including the mitogen-activated protein kinases (MAPKs) and the IκB kinases (IKKs). Activation of the IKKs induces phosphorylation of the IκBs, which leads to their subsequent degradation through ubiquitin-mediated pathways. The degradation of IκBs reveals the nuclear localization signals in NF-κB; dimerization and nuclear accumulation of combinations of NF-κB subunit proteins ensues, thus facilitating signal-mediated regulation of gene transcription within a given cell type, including those that contribute to inflammatory responses [20], [22]. The CCL2 gene contains NF-κB response elements in its proximal gene promoter and is responsive to IL-1β and other stimuli [23], [24]. However, the transcription factors and associated signaling pathways responsible for controlling expression of CCL2 in pancreatic β-cells have not been established.

Signaling through the MAPKs often links extracellular signals to specific gene promoters [25]. For example, the p38 MAPK is linked to inflammation in multiple tissues, including the pancreatic β-cell [26], [27] and systemic inhibition of p38 delays diabetes progression in the non-obese diabetic (NOD) mouse [28]. Thus, strategies to downregulate p38 MAPK may be a therapeutic approach to prevent chemokine release and subsequent immune cell recruitment. The MAPK phosphatases, a subset of the family of dual specificity phosphatases (DUSPs), could be one such targetable approach. The genes encoding several of these phosphatases are regulated by glucocorticoids (GCs) in a variety of tissues [29], [30], [31].

GCs are often used in a variety of clinical situations to decrease inflammation. These steroids activate the intracellular glucocorticoid receptor (GR), leading to suppression of many outcomes controlled by the NF-κB pathway [32]. GR activation coordinately alters transcriptional patterns within many different cell types, leading to both activation and repression of a multitude of genes, and shifts cellular phenotype towards an anti-inflammatory state [32], [33]. MKP-1 (also known as DUSP1) gene transcription is increased by glucocorticoids [29], [34]. The increase in MKP-1 protein promotes p38 MAPK dephosphorylation, thus diminishing stimulus-specific kinase activity. Since p38 MAPK integrates cell surface receptor-mediated signaling pathways to inflammatory responses [27], upregulation of MKP-1 in pancreatic β-cells may represent a viable strategy to decrease inflammation-associated pathologies, such as cytokine-mediated increases in chemokine production.

We undertook this study to investigate the regulation of the CCL2 gene by IL-1β and GCs in rat islets and β-cell lines. We hypothesized that expression of the MKP-1 gene, which we show is induced by multiple GCs in rat islets and β-cell lines, would provide anti-inflammatory actions that decrease expression of the CCL2 gene. We discovered that augmenting MKP-1 levels partially mimicked the inhibition of the IL-1β-mediated increase in CCL2 gene expression and secretion seen with GCs. Importantly, the anti-inflammatory actions associated with MKP-1 overexpression were obtained without suppressing glucose-stimulated insulin secretion, demonstrating that this gene is a possible target for therapeutic intervention with preservation of β-cell function.

## Experimental Procedures

### Cell Culture, Islet Isolation, Glucose-stimulated Insulin Secretion, and Reagents

The establishment of the 832/13 and INS-1E rat insulinoma cells has been described [35], [36]. These cell lines were maintained in RPMI-1640 (Mediatech; Manassas, VA) with 10% fetal bovine serum (FBS; Life Technologies Co., Carlsbad, CA). Islets were isolated from Wistar rats as outlined in prior studies [37]. All animal experiments were conducted in accordance with Duke University IACUC guidelines using approved procedures in appropriately accredited facilities (protocol #: A309-10-12). Buffer components and procedures for measuring insulin release into the media following secretagogue exposure were performed as previously published [35]. IL-1β was from Thermo Fisher Scientific (Waltham, MA) and γ-IFN was purchased from Shenandoah Biotechnology Inc. (Warwick, PA). Dexamethasone, hydrocortisone, budesonide, fluticasone propionate, TPCA, and all MAPK inhibitors used herein were from Tocris Bioscience (Ellisville, MO). Recombinant adenoviruses expressing 5X NF-κB-luciferase [38], β-Galactosidase [39], p65 wild-type and S276A [40], IκBα super-repressor [41] and MKP-1 [42] have all been described. The CCL2-luciferase reporter [24], the 3X GAS-luciferase reporter [43], and the MKP-1-luciferase reporter constructs [34] have also been documented. The adenovirus expressing hIKKβ S177E/S181E was a kind gift from Dr. Haiyan Xu (Brown University).

### Isolation of RNA, cDNA Synthesis and Real-time RT-PCR

Total RNA was isolated using Isol-RNA Lysis Reagent (5 Prime Inc, Gaithersburg, MD), cDNA synthesized and real-time RT-PCR performed using SYBR Green (Applied Biosystems, Carlsbad, CA) as previously described [38]. Primers for COX2, CCL2, MKP-1, and RS9 used to detect transcript levels via RT-PCR reactions are available upon request.

### Isolation of Protein and Immunoblot Analysis

Whole cell lysates were prepared using M-PER (Thermo Fisher Scientific) supplemented with protease and phosphatase inhibitor cocktails (Thermo Fisher Scientific). Proteins were quantified using the BCA method (Thermo Fisher Scientific). SDS-PAGE, transfer to PVDF, and blocking of membranes prior to antibody incubation as well as subsequent downstream detection has been described [44]. Antibodies used in this study were from the following sources: MKP-1 was from Santa Cruz Biotechnology, Inc. (Santa Cruz, CA), while PO_4_− and total p38, PO_4_− and total JNK, PO_4_− and total ERK, as well as p65 were all from Cell Signaling Technology, Inc. (Danvers, MA). Anti-β Actin and anti-FLAG were from Sigma Aldrich.

### Plasmid and siRNA Transfection and Luciferase Assays

Transient transfections of plasmids and siRNA duplexes into 832/13 cells and cell lysis for luciferase assays were as described [45]. Reporter gene activity was analyzed as previously described [46]. The Silencer Select siRNA duplexes were obtained from Life Technologies Co.(siRNA ID for p65: s159517; siRNA ID for MKP-1: s137873) and negative control siRNA sequence (catalog no. M4611). Delivery was via Dharmafect reagent 1 (Dharmacon, Lafayette, CO) according to the manufacturer's protocol for duplexes and FuGene6 for plasmids. Dharmafect Duo (Dharmacon) was used in all experiments requiring simultaneous delivery of plasmids and siRNA duplexes.

### ELISA

Detection of CCL2 secreted into the media was performed using the Quantikine kit from R & D Systems, Inc. (Minneapolis, MN) according to their suggested protocol. Chemokine release into the media was normalized to total protein to account for any potential differences in cell number.

### Immunofluorescence

832/13 cells were seeded onto glass cover slips within a 6-well plate. Following treatments described in the Figure Legends, the cells were fixed using 4% paraformaldehyde for 20 min, followed by PBS washing three times, five minutes each time. After the wash steps, glass cover slips were moved to a humidified chamber and exposed to a blocking solution containing 10% Goat Serum (Sigma, St. Louis, MO), 0.25% Triton X-100, and 1X PBS for 1 h at room temperature. After this blocking incubation, the blocking solution was removed by aspiration and the primary antibody [p65 (Santa Cruz Catalog Number sc-372)] diluted in 10% Goat Serum, 0.25% Triton X-100, PBS was applied and left overnight at 4°C. After approximately 16 h of incubation with primary antibody, the cells were again washed with PBS three times for five minutes each time. After washing with PBS, cover slips were returned to the humidified chamber and incubated with secondary antibody (Alexa Fluor® 488 goat anti-rabbit IgG (H+L) Invitrogen Molecular Probes A11008) for 1 h at room temperature. After the secondary antibody incubation, cover slips were washed with PBS – three times for five minutes each time. After washes were complete, cover slips were mounted onto glass slides using Prolong Gold w/DAPI Anti-fade reagent (Life Technologies Co.). These slides were analyzed by epifluorescence microscopy (Nikon Eclipse Ti-E) using a 60x objective lens (NA 1.49). 3×3 large image scans were collected and analyzed using NIS-Elements AR v3.1 software (Nikon Instruments, Melville, NY).

### Isolation of Nuclear Protein and Electrophoretic Mobility Shift Assays

Subcellular fractionation and isolation of nuclear protein from 10 cm dishes was performed using the NE-PER kit from Thermo Scientific exactly as directed by the manufacturer’s protocol. Oligonucleotides corresponding to the NF-κB sequences present in the rat CCL2 gene promoter were synthesized with 5′-biotin labels, purified by HPLC, and then annealed prior to incubation with 10 µg of nuclear protein. The sequence of the sense oligo (NF-κB sites underlined) is: 5′-ggtctgggaacttccaatactgcctcagaatgggaatttccacact- 3′. Cold competitor oligos (i.e., without biotin tags) were synthesized and purified in the same manner. The binding reactions were set up as described in the LightShift Chemiluminescent EMSA kit (Thermo Scientific). These reactions were then separated on 6% DNA retardation gels (Invitrogen), followed by transfer to nylon membranes. The protein-DNA complexes were crosslinked to the nylon membrane by using a UV transilluminator with 312 nm bulbs. Blocking of the membrane and subsequent downstream detection of the biotin label were exactly as described in the LightShift EMSA kit protocol.

### Statistical Analysis

One way ANOVA analysis with Tukey’s post-hoc correction was performed with statistical significance at the 90%, 95% and 99% confidence intervals denoted in the figure legends.

## Results

### CCL2 mRNA Abundance is Increased by Pro-inflammatory Cytokines in Rat Islets and β-cell Lines

**Figure 1:**
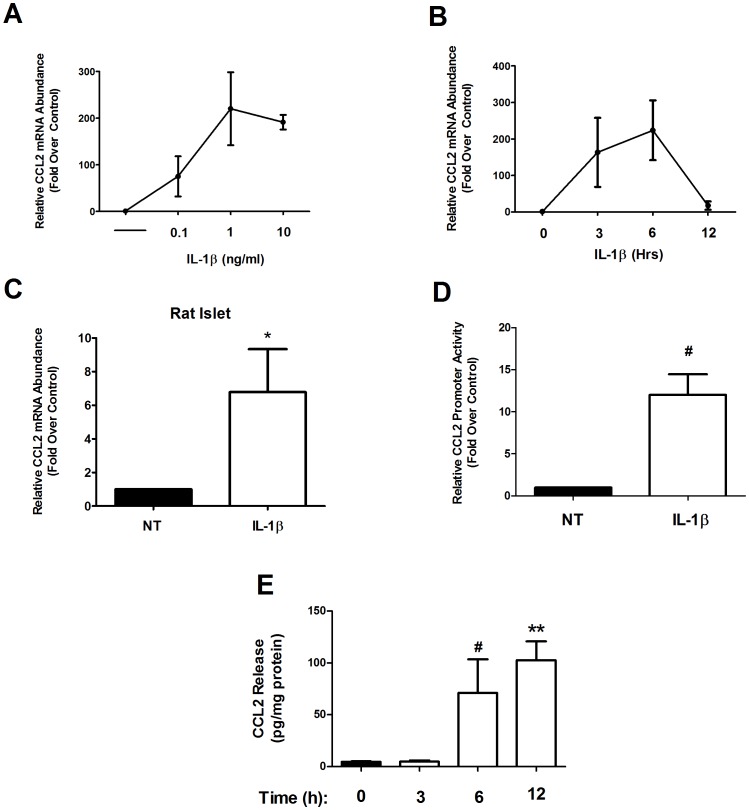
CCL2 mRNA accumulation is induced by pro-inflammatory cytokines in rat islets and β-cell lines. *A*. 832/13 rat insulinoma cells were treated with 0.1, 1, and 10 ng/mL of IL-1β for 6 h. *B*. 832/13 cells were stimulated with 1 ng/mL IL-1β for 0, 3, 6 and 12 h. *C*. Rat islets were either untreated or treated with 10 ng/mL IL-1β for 6 h. *A–C*. Total RNA was isolated and CCL2 mRNA levels were measured and normalized to those of the housekeeping gene, Ribosomal S9 (RS9). *p<0.05 vs. NT. *D*. 832/13 cells were transfected with 3.6 kb of the CCL2 promoter upstream of the transcriptional start site fused to a luciferase reporter (3.6-Luc). 24 h post-transfection cells were stimulated for 4 h with 1 ng/mL IL-1β. Relative promoter activity of 3.6-Luc was measured and normalized to protein content via BCA assay. **^#^** *p*<0.001 vs. NT. *E*. 832/13 cells were untreated or treated with 1 ng/mL IL-1β for 0, 3, 6 and 12 h. CCL2 release into the media was measured by ELISA and was normalized to protein content via BCA assay. **^#^** *p*<0.001 vs. 0 h, **p<0.01 vs. 0 h. Data are means ± SEM from 3–4 individual experiments.

CCL2 co-localizes with insulin-positive cells in the pancreatic islets [47], [48]. In addition, the CCL2 gene promoter contains genomic elements, such as NF-κB and gamma activated sequences (GAS), which indicate potential responsiveness to pro-inflammatory cytokines. We therefore examined the effects of IL-1β and γ-IFN, two distinct pro-inflammatory cytokines involved in β-cell death and dysfunction [49], [50], on the expression of this gene using rat islets and β-cell lines. In 832/13 rat insulinoma cells, we observed that 1 ng/mL IL-1β is sufficient to drive maximal expression of the CCL2 gene, as increasing the amount 10-fold to 10 ng/mL does not promote additional mRNA accumulation (Figure 1A). Next, we examined CCL2 mRNA accumulation over time, noting that expression increased within 3 h, was maximal at 6 h, and then decreased by 12 h after exposure to 1 ng/mL IL-1β (Figure 1B). Expression of the CCL2 gene is also enhanced by IL-1β in isolated rat islets (Figure 1C). We note that while there is a GAS response element present in the CCL2 gene promoter, γ-IFN had no effect on the expression of the CCL2 gene in 832/13 cells while in rat islets, there was a small response (∼20% of that produced by IL-1β; not shown). By contrast, transcription from 3.6 kb of the proximal CCL2 gene promoter linked to a luciferase reporter was strongly increased in response to IL-1β (Figure 1D). This transcriptional readout correlated with augmented secretion of CCL2 protein over time (Figure 1E), indicating that expression of the gene is most likely coupled to secretion of protein. While we observed low levels of CCL2 gene expression in the basal state (e.g., without IL-1β exposure), there was a marked induction in response to IL-1β (cycle threshold (Ct) values are given in Figure S1).Thus, we conclude that IL-1β drives the synthesis and secretion of CCL2 in rat pancreatic β-cells.

### The p65 Subunit of NF-κB is Both Necessary and Sufficient for Expression of the CCL2 Gene

**Figure 2:**
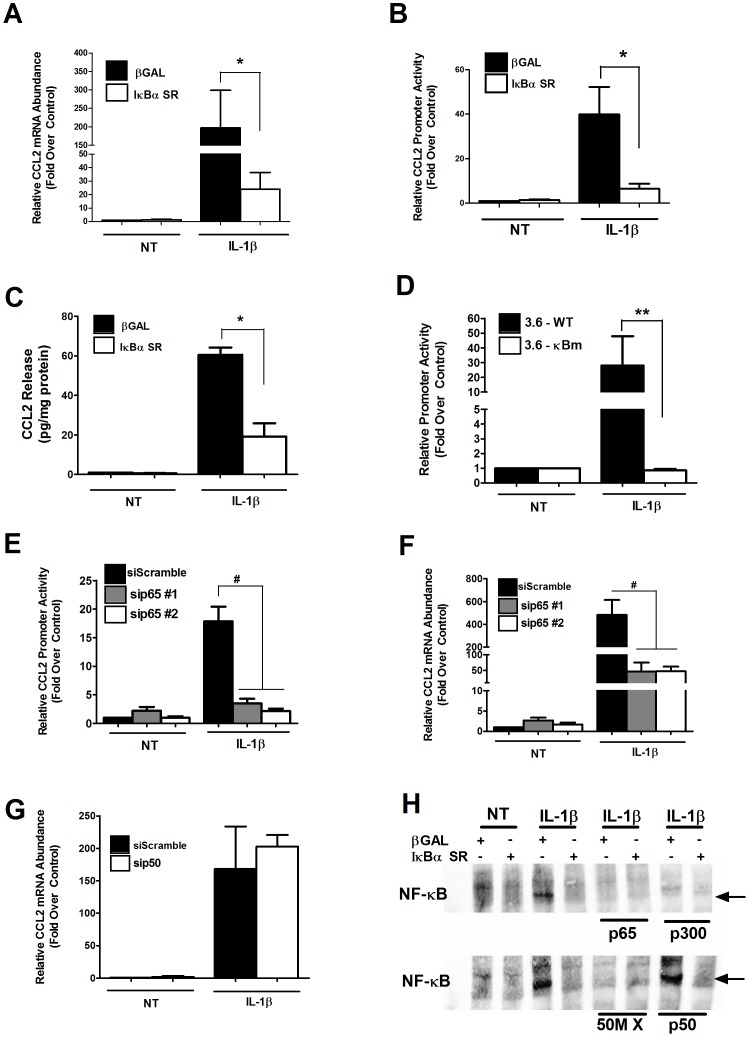
The p65 subunit of NF-κB is required for IL-1β- mediated expression of the CCL2 gene. *A*. 832/13 cells were transduced with adenoviruses expressing either β-Galactosidase (βGAL) or IκBα S32A/S36A for 24 h followed by stimulation with 1 ng/mL IL-1β for 6 h. *p<0.05. *B*. Cells were transduced with the indicated adenoviruses for 24 h, transfected with 3.6-Luc promoter construct for 4 h, followed by a 4 h stimulation with IL-1β. *p<0.05. *C*. 832/13 cells were transduced with the indicated adenoviruses; 24 h post-transduction cells were treated with IL-1β for 12 h. CCL2 release into the media was measured by ELISA and was normalized to protein content via BCA assay. *p<0.05. *D*. 832/13 cells were transfected with either 3.6-Luc (3.6 -WT) or 3.6–κBm; 24 h post-transfection cells were treated with IL-1β for 4 h. **p<0.01. *E.* 832/13 cells were co-transfected with either two different siRNA duplexes targeting p65 and 3.6-Luc promoter construct. 24 h post-transfection cells were treated with 1 ng/mL IL-1β for 4 h. **^#^** *p*<0.001. *F*. 832/13 cells were transfected with two duplexes against p65 using a scrambled siRNA sequence duplex as a control. 24 h post- transfection cells were treated for 6 h with IL-1β. **^#^** *p*<0.001. *A, F*. Total RNA was isolated, and RT-PCR was performed using CCL2 primers; expression of CCL2 was normalized to RS9. *B, D, E.* Promoter activity was analyzed via luciferase assay and values normalized to protein content using a BCA assay. *G*. DNA binding using nuclear extracts from cells treated as indicated on the top of the image. The specific antisera or cold-competitor oligos are indicated on the bottom of the image. The arrow indicates the specific complex bound to the NF-κB DNA sequence from the CCL2 gene promoter. All data in A–F represent means ± SEM from 3–4 individual experiments, while the image in G. is representative of 2–3 experiments. 50M X, 50-fold molar excess of unlabeled (cold) oligonucleotide.

We next directly manipulated the p65 subunit of NF-κB to determine its involvement in the regulation of CCL2 gene expression by IL-1β. First, we used the IκBα super-repressor, which contains two amino acid substitutions (S32A/S36A) that eliminate phosphorylation-induced degradation of the regulatory protein in response to pro-inflammatory stimuli [41]. The NF-κB subunits are normally released from regulatory subunits, such as IκBα, for translocation to the nucleus after phosphorylation-induced regulatory subunit degradation [20]. Thus, the mutant IκBα protein attenuates NF-κB pathway actions by retaining p65 in the cytoplasm [41]. We observed that overexpression of the IκBα super-repressor blocked the IL-1β-mediated increases in CCL2 mRNA by 79%, promoter activity by 84%, and secretion by 68% (Figure 2 A–C).

We then compared the wild-type CCL2 gene promoter (3.6– WT) to a construct that contains point mutations abolishing the known NF-κB binding sites (3.6–κBm) (Figure 2D). The NF-κB mutant reporter construct was refractory to stimulation by IL-1β, while the wild-type construct was robustly responsive (28-fold; Figure 2D). Since the wild-type CCL2 gene promoter is strongly induced by IL-1β, we further examined which NF-κB subunits were required for transcriptional induction in response to cytokines. Using siRNA-mediated suppression of p65 (76% decrease in mRNA - not shown) and corresponding decrease in both cytoplasmic and nuclear protein (Figure S2), we discovered that the CCL2 gene requires p65 for IL-1β-mediated transactivation (Figures 2E–F).

We next investigated binding to the NF-κB element present within the CCL2 proximal gene promoter using electrophoretic mobility shift assays. We discovered that NF-κB bound to this genomic region was detected only using nuclear fractions under IL-1β stimulated conditions (Figure 2G). Furthermore, the complex associated with the NF-κB response elements was abrogated by overexpressing the IκBα super-repressor construct (Figure 2G). To address specificity of the bound complex, we demonstrated that inclusion of fifty-fold molar excess (50 M X) of unlabeled competitor oligo diminished the specific protein-DNA complex (Figure 2G, bottom). Moreover, the particular NF-κB complex associated with the CCL2 genomic response element was impaired in the presence of antisera targeted against either p65 or the coactivator p300, but not with antisera against p50 (Figure 2G). The DNA binding data is thus consistent with NF-κB protein requirements for activation of the CCL2 gene (see Figures 2 E–G). Collectively, we interpret these results to indicate that the IL-1β-mediated activation of the CCL2 gene in pancreatic β-cells requires the p65 subunit of NF-κB.

**Figure 3:**
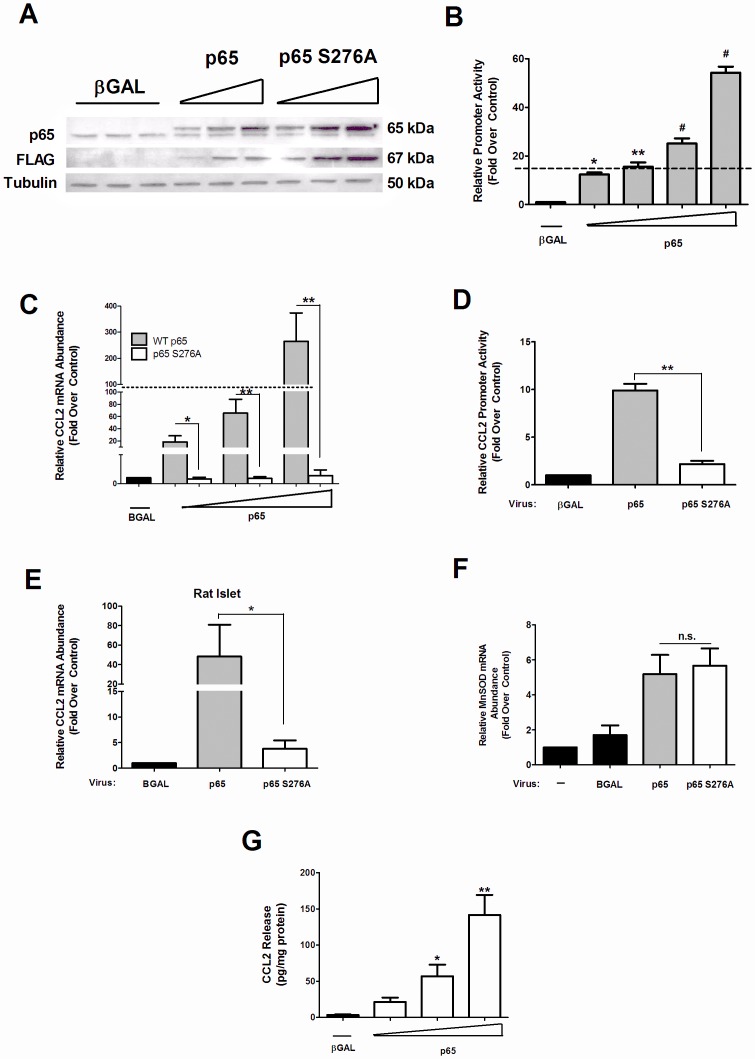
p65 overexpression is sufficient for activation of the CCL2 gene in rat islets and β-cell lines. *A*. 832/13 cells were transduced with increasing concentrations of recombinant adenoviruses expressing either βGAL, FLAG-p65 and FLAG-p65 S276A. Whole cell lysates were harvested 24 h post-transduction and an immunoblot was performed using antibodies against p65 (detecting both endogenous and ectopically overexpressed protein), FLAG (only detects virally-produced form of the p65 protein) and tubulin (as a control for protein loading). The blot shown is representative of two independent experiments. *B–G.* Rat islets (E) and 832/13 cells (B, C, D, F, G) were treated for 24 h with the indicated adenoviruses. *B, D.* 4 h following adenoviral transduction cells were transfected with either a 5X NF-κB- Luc promoter (B) or the 3.6-Luc promoter (D) for 24 h; promoter activity was normalized to protein content. *B*. *p<0.05 vs. βGAL; **p<0.01 vs. βGAL; **^#^** *p*<0.001 vs. βGAL, D,**p<0.01 vs. p65 S276A. *C, E* and *F*. Total RNA was isolated and gene expression monitored via real-time PCR. *C*. *p<0.05 vs. p65 S276A; **p<0.01, *E*. *p<0.05, *F.* n.s. = not significant. *G*. CCL2 secretion into the cell culture media was measured via ELISA and the data normalized to total intracellular protein content. *p<0.05 vs. βGAL; **p<0.01 vs. βGAL. RNA abundance, promoter luciferase activity, and ELISA data are represented as means ± SEM from 3–4 individual experiments. The dashed line in B. and C. represents the induction by IL-1β exposure.

Because p65 is *necessary* for the IL-1β-mediated induction of the CCL2 gene (Figure 2E and F), we next examined whether p65 overexpression was *sufficient* to drive the expression of the CCL2 gene. Figure 3A shows the increase in p65 abundance produced by the viral transgene. This increase in p65 protein driven by the viral transgene enhanced the expression of a synthetic multimerized NF-κB luciferase gene in a dose-dependent fashion (Figure 3B; dashed line indicates promoter activity induced by 1 ng/mL IL-1β). Next, we examined the expression of the CCL2 gene in response to overexpression of p65 and observed an 18.5, 53.6 and 215.2-fold enhancement in mRNA levels (Figure 3C), demonstrating that simply enhancing p65 abundance is also sufficient to increase the expression of the gene in a dose-dependent manner. By contrast, removal of a phosphoacceptor site at Ser276 within p65, which is known to control recruitment of the CBP/p300 co-activators, diminished the ability of p65 to drive expression of the CCL2 gene (Figure 3C) and 3.6 kb of the proximal CCL2 gene promoter (Figure 3D). The expression levels of CCL2 driven by p65 overexpression are similar to the effect seen with IL-1β exposure (note that the dashed line in Figure 3B and C represents the induction by IL-1β). We observed that while the 3.6 kb promoter construct containing mutations within the NF-κB elements is unresponsive to p65 overexpression (data not shown), transcription of the wild-type CCL2 gene promoter is markedly enhanced by this maneuver (Figure 3D). By contrast, the COX2 gene, which requires p65 for induction by IL-1β [38], is not activated simply by p65 overexpression (data not shown). Similar results were obtained in isolated rat islets (Figure 3E). Importantly, expression of the manganese superoxide dismutase (MnSOD) gene was not different between the wild-type and S276A forms of p65 (Figure 3F), demonstrating that both wild-type p65 and the S276A mutant proteins are transcriptionally competent in the β-cell. Finally, we detected a dose-dependent increase in secretion of CCL2 protein with overexpression of p65 (Figure 3G). Therefore, we conclude that augmenting p65 abundance was sufficient to drive the expression of the CCL2 gene, which resulted in secretion of CCL2 protein.

### IKKβ Drives the Expression of the CCL2 Gene

**Figure 4:**
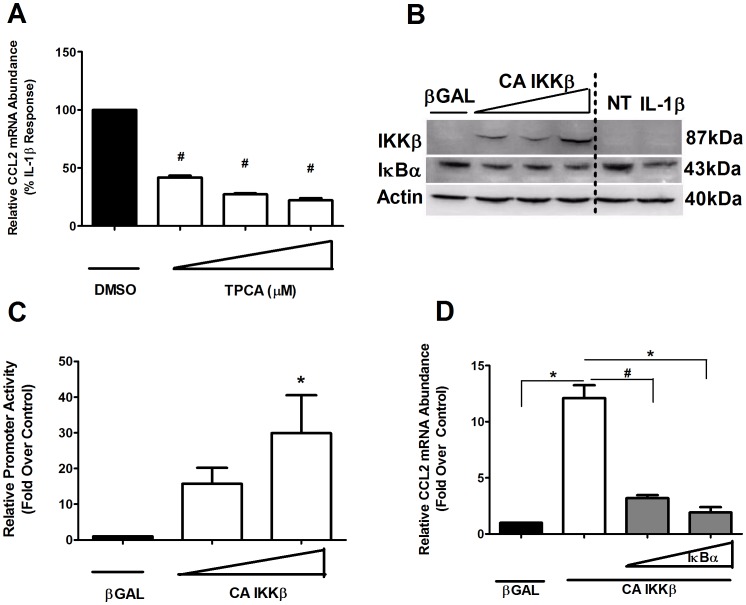
IKKβ drives the expression of the CCL2 gene. *A*. 832/13 cells were pre-treated for 1 h with 0.5, 1 or 2 µM TPCA, followed by a 6 h stimulation with 1 ng/mL IL-1β. **^#^** *p*<0.001 vs. DMSO. *B*. Cells were transduced with either βGAL or three increasing concentrations of IKKβ S177E/S181E (CA IKKβ) overnight, or 1 ng/mL IL-1β for 15 mins (dashed line indicates separation between cytokine-stimulated IκBα degradation from that induced by CA-IKKβ). Whole cell lysates were harvested and immunoblot analysis was performed using antibodies to detect IKKβ, IκBα and β Actin. *C.* Cells were transduced with the indicated adenoviruses and subsequently transfected with 5X NF-κB-Luc; relative promoter activity was normalized to total cellular protein. *p<0.05 vs. βGAL. *D.* Cells were treated with either CA-IKKβ adenovirus alone or in the presence of two increasing concentrations of IκBα SR. Suppression by the IκBα SR is shown in the grey bars. *p<0.05 vs. βGAL**;^ #^** *p*<0.001 vs. respective controls. *A, D.* Total RNA was isolated and CCL2 gene expression monitored via real-time PCR. Promoter luciferase activity and RNA abundance data represent means± SEM from 3–4 individual experiments.

We next examined specific signaling pathways responsible for activation of the CCL2 gene. Using 2-[(Aminocarbonyl)amino]-5-(4-fluorophenyl)-3-thiophenecarboxamide (TPCA), a pharmacological inhibitor of IKKβ, we found that the ability of IL-1β to induce expression of the CCL2 gene was diminished by 57, 73 and 77% (Figure 4A). To further demonstrate the involvement of IKKβ, we overexpressed a constitutively-active (S177E/S181E) form of the kinase (CA-IKKβ) and examined the degradation of the IκBα protein, a known target of IKKβ. We detected a decrease in IκBα protein abundance in the present of the CA-IKKβ (Figure 4B; left side of dashed line), which was similar to the degradation induced by IL-1β (Figure 4B; right side of dashed line). Accordingly, we discovered that expression of the CA-IKKβ increased the transcriptional activity of a synthetic NF-κB reporter gene by 15.7 and 29.9-fold (Figure 4C). Examination of CCL2 mRNA levels revealed a 15-fold increase in the presence of the CA-IKKβ (Figure 4D). To ensure specificity for NF-κB-mediated induction of the CCL2 gene by IKKβ, we combined overexpression of the IκBα super-repressor with CA-IKKβ expression; this experiment revealed a 73% and 84% reduction in the ability of CA-IKKβ to induce the expression of CCL2 (Figure 4D). Thus, we demonstrate for the first time in pancreatic β-cells that IKKβ is a key participant in the regulation of the CCL2 gene by NF-κB.

### The Expression of the CCL2 Gene is Sensitive to Inhibition of the p38 MAPK

**Figure 5:**
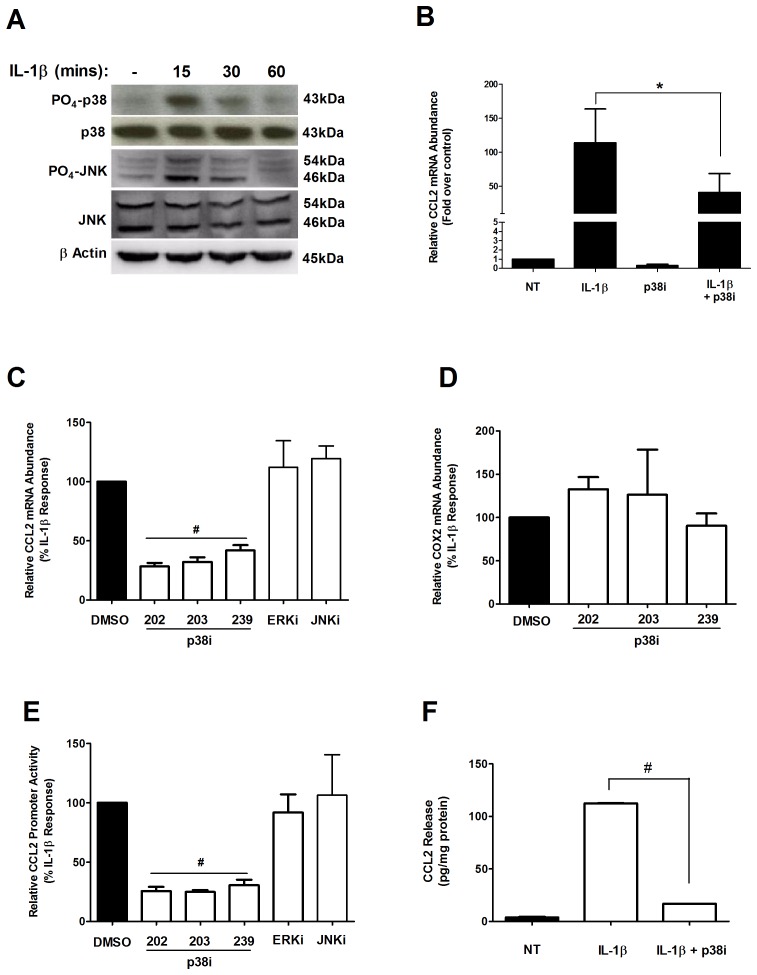
The expression of the CCL2 gene is sensitive to p38 MAPK inhibition. *A*. 832/13 cells were treated for 15, 30 or 60 min with 1 ng/mL IL-1β. Immunoblot analysis was performed on three separate occasions with the indicated antibodies and a representative image is shown. *B*. Rat islets were pre-treated for 1 h with 10 µM of SB202190 (p38i = p38i inhibitor) followed by a 6 h incubation with 10 ng/ml IL-1β. *p<0.05. *C, D*. 832/13 cells were pre-treated for 1 h with 10 µM of SB202190, SB203580, SB239063, 1 µM SP600125 (JNK inhibitor; JNKi), or 20 µM PD98059 (ERK inhibitor; ERKi) followed by a 6 h incubation with 1 ng/mL IL-1β. Steady-state mRNA abundance for CCL2 and COX2 was quantified and normalized to RS9. **^#^** *p*<0.001 vs. DMSO *E*. 832/13 cells were transfected with 3.6-Luc; 24 h post-transfection cells were pre-treated for 1 h with the respective MAPK inhibitors (10 µM p38 inhibitors, 20 µM ERKi or 1 µM JNKi), followed by a 4 h incubation with 1 ng/mL IL-1β. Luciferase reporter activity was normalized to protein content. **^#^** *p*<0.001 vs. DMSO. *F*. 832/13 cells were pre-treated for 1 h with 10 µM SB202190, followed by 12 h stimulation with 1 ng/mL IL-1β. An ELISA was performed to determine CCL2 release into the media, and results were normalized to total protein content. **^#^** *p*<0.001. RNA abundance, luciferase promoter activity and ELISA data are expressed as means ± SEM from 3–4 individual experiments.

IL-1β activation of the IL-1 receptor is linked to a number of different signaling pathways, including the IKKs (see [20] and Figure 4 above) and the MAPKs [51]. To further examine the specific signaling pathways involved with IL-1β-mediated increases in the expression of the CCL2 gene in pancreatic β-cells, we examined phosphorylation of p38, JNK, and ERK in 832/13 cells. Both JNK and p38 were rapidly phosphorylated (within 15 min) upon IL-1β exposure (Figure 5A). By contrast, we found that ERK is not activated in response to IL-1β out to 60 mins (data not shown). The lack of ERK phosphorylation by IL-1β in pancreatic β-cells observed in our study is consistent with a prior report [52].

We next used pharmacological inhibitors that interfere with p38 kinase activity to determine if this maneuver hindered IL-1β-mediated increases in CCL2 gene expression and promoter activity. We discovered that pharmacological inhibition of p38 by SB202190 diminished the IL-1β-induced expression of the CCL2 gene in rat islets by 65% (Figure 5B). Moreover, in 832/13 cells, each of the pyridinyl imidazole based p38 inhibitors used - SB202190, SB203580, and SB239063 - markedly inhibited the IL-1β-stimulated expression of the CCL2 gene (Figure 5C). Although JNK is phosphorylated in response to IL-1β, blocking JNK activity using SP600125 (JNKi) had no impact on IL-1β- mediated induction of the CCL2 gene (Figure 5C); however, this inhibitor decreased expression of the GADD45 gene (Figure S3). Similarly, inhibition of ERK activity using 20 µM PD98059 did not impair IL-1β- mediated expression of the CCL2 gene (Figure 5C); these findings are congruent with our lack of observed ERK phosphorylation in response to IL-1β in pancreatic β-cells. The findings described herein are also consistent with the lack of ERK phosphorylation by IL-1β in a previous study [52]. Furthermore, the ERK inhibitor did blunt the γ-IFN-mediated induction of a multimerized GAS element driving the luciferase gene (Figure S4). While IL-1β induced an 8-fold increase (set at 100%) in the expression of the COX2 gene, this induction was unaffected by inhibition of the p38 MAPK (Figure 5D). In addition to the results observed in Figures 5B & 5C, the transcriptional activity of the CCL2 promoter in response to IL-1β was also diminished by pharmacological inhibition of p38 (Figure 5E). Moreover, the IL-1β-mediated increase in CCL2 secretion was reduced by 85% in the presence of p38 inhibitor SB202190 (Figure 5F). We interpret these data collectively to indicate that while the CCL2 and COX2 genes are both NF-κB responsive, there are specific and distinct signaling inputs required for their expression in the pancreatic β-cell in response to IL-1β.

### Activation of the Glucocorticoid Receptor Suppresses IL-1β-mediated Induction of the CCL2 Gene

**Figure 6:**
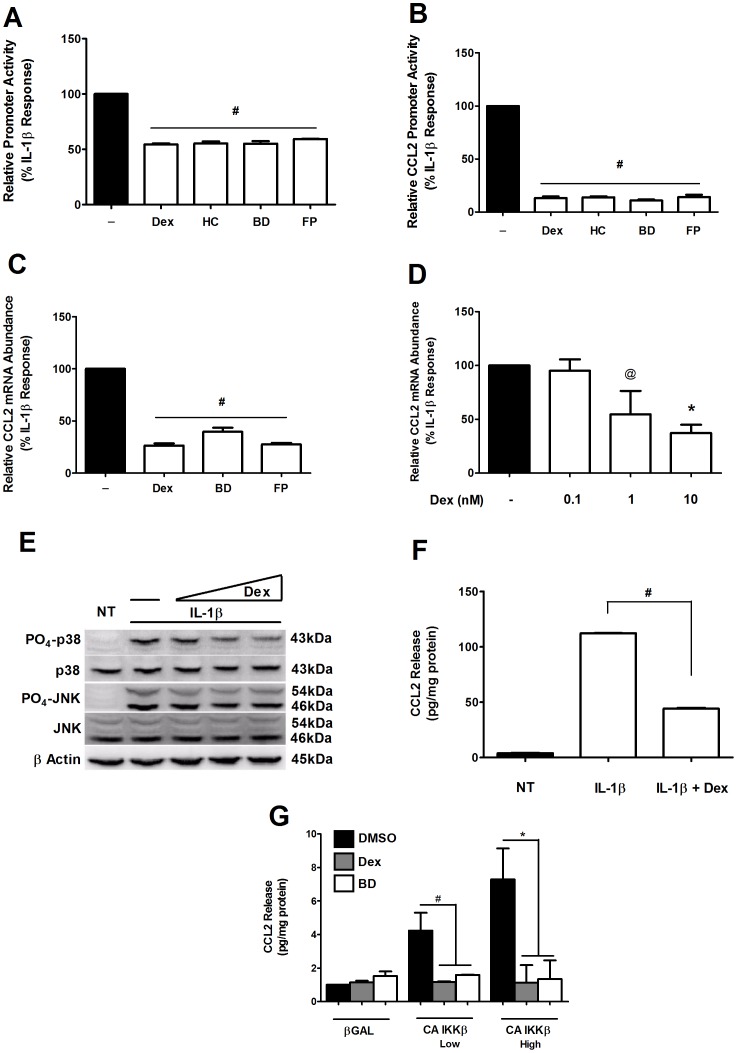
Activation of the glucocorticoid receptor suppresses IL-1β-mediated induction of the CCL2 gene. *A,B.* 832/13 cells were transfected with 5X NF-κB-Luc (A) or 3.6-Luc (B) followed by co-treatment with either 10 nM Dexamethasone (Dex), 100 nM Hydrocortisone (HC), 10 nM Budesonide (BD) or 10 nM Fluticasone Propionate (FP) and 1 ng/mL IL-1β for 4 h. Luciferase promoter activity was quantified and normalized to total cellular protein. **^#^** *p*<0.001 vs. DMSO (black bar). *C*. 832/13 cells were co-stimulated for 6 h with 10 nM Dex, BD or FP in the presence of 1 ng/mL IL-1β. *D*. 832/13 cells were treated with increasing concentrations of Dex (0.1, 1, 10 nM) for 6 h in the presence of 1 ng/mL IL-1β. *C, D*. CCL2 mRNA accumulation was assessed via RT-PCR and normalized to RS9 mRNA abundance. **^#^** *p*<0.001 vs. DMSO., **^@^** *p*<0.1 vs. DMSO, *p<0.05 vs DMSO. *E*. 832/13 cells were pre-treated with 0.1, 1 or 10 nM Dex for 6 h followed by 15 min stimulation with IL-1β. Immunoblot analysis was performed with antibodies against p38, PO_4_-p38, JNK, PO_4_-JNK and β Actin. *F*. ELISA was used to quantify CCL2 release into the media following a12 h incubation with media alone, media containing 1 ng/mL IL-1β or 1 ng/mL IL-1β plus 10 nM Dex. **^#^** *p*<0.01. *G*. 832/13 cells were transduced with recombinant adenoviruses expressing either βGAL or increasing concentrations of CA IKKβ for 12 h in the presence of either 10 nM Dex or BD. CCL2 release into the media was quantified using an ELISA. **^@^** *p*<0.1, *p<0.05. The bar graphs represent means ± SEM from 3–4 individual experiments.

After discovering that the p38 MAPK was required for activation of the CCL2 gene by IL-1β, we next tested whether anti-inflammatory mechanisms targeting this pathway could block this induction. Because glucocorticoids are often used clinically for their potent anti-inflammatory properties, we treated 832/13 cells with a panel of known glucocorticoid receptor ligands: dexamethasone (Dex), hydrocortisone (HC), budesonide (BD), and fluticasone propionate (FP). As shown in Figure 6A, each GR agonist strongly inhibited the ability of IL-1β to induce the transcription of a multimerized NF-κB luciferase reporter gene. The effect was even more striking on the IL-1β-mediated activation of the 3.6 kb proximal CCL2 luciferase reporter gene (Figure 6B).

We next observed that the IL-1β-mediated increase in CCL2 mRNA levels was diminished by 74%, 61%, and 73% by Dex, BD, and FP, respectively (Figure 6C). To begin to address the mechanism of GC-mediated repression, we examined the sensitivity of the CCL2 gene to Dex. We observed decreases of 5%, 46%, and 63% in CCL2 mRNA levels in response to increasing increments in dexamethasone concentration (Figure 6D). Dex exposure also diminished the IL-1β-mediated phosphorylation of the p38 MAPK (Figure 6E). In turn, this resulted in a 60% decrease in secreted CCL2 (Figure 6F), which is congruent with the results in Figure 5. Furthermore, while CCL2 secretion was driven by the constitutive-active IKKβ, release of peptide was markedly repressed by both Dex and BD (Figure 6G). Thus, these results are consistent with a role for IKKβ and p38 MAPK to induce expression and secretion of CCL2 and demonstrate further that synthetic glucocorticoids interrupt this process, likely by interfering with the signaling mechanisms used by IL-1β.

Because Dex blocks p65 nuclear translocation in mast cells [53], we next tested the hypothesis that glucocorticoids have a similar function in β-cells, which could explain the repressive effects on CCL2 gene expression. Using 832/13 cells, IL-1β stimulated the nuclear translocation of p65 to a similar level in both the presence and absence of Dex (Figure S5). Thus, the immunofluorescence assays reveal that in contrast to the mast cell, where Dex prevents nuclear translocation of p65 [53], no such blockade in trafficking exists in glucocorticoid-treated 832/13 rat insulinoma cells that are also exposed to IL-1β. We therefore conclude that the repressive actions of GCs in pancreatic β-cells, such as seen in Figure 6 A–G must exist through mechanisms other than cytoplasmic retention of p65.

### Induction of the MKP-1 Gene by Glucocorticoids Promotes Dephosphorylation of the p38 MAPK

**Figure 7:**
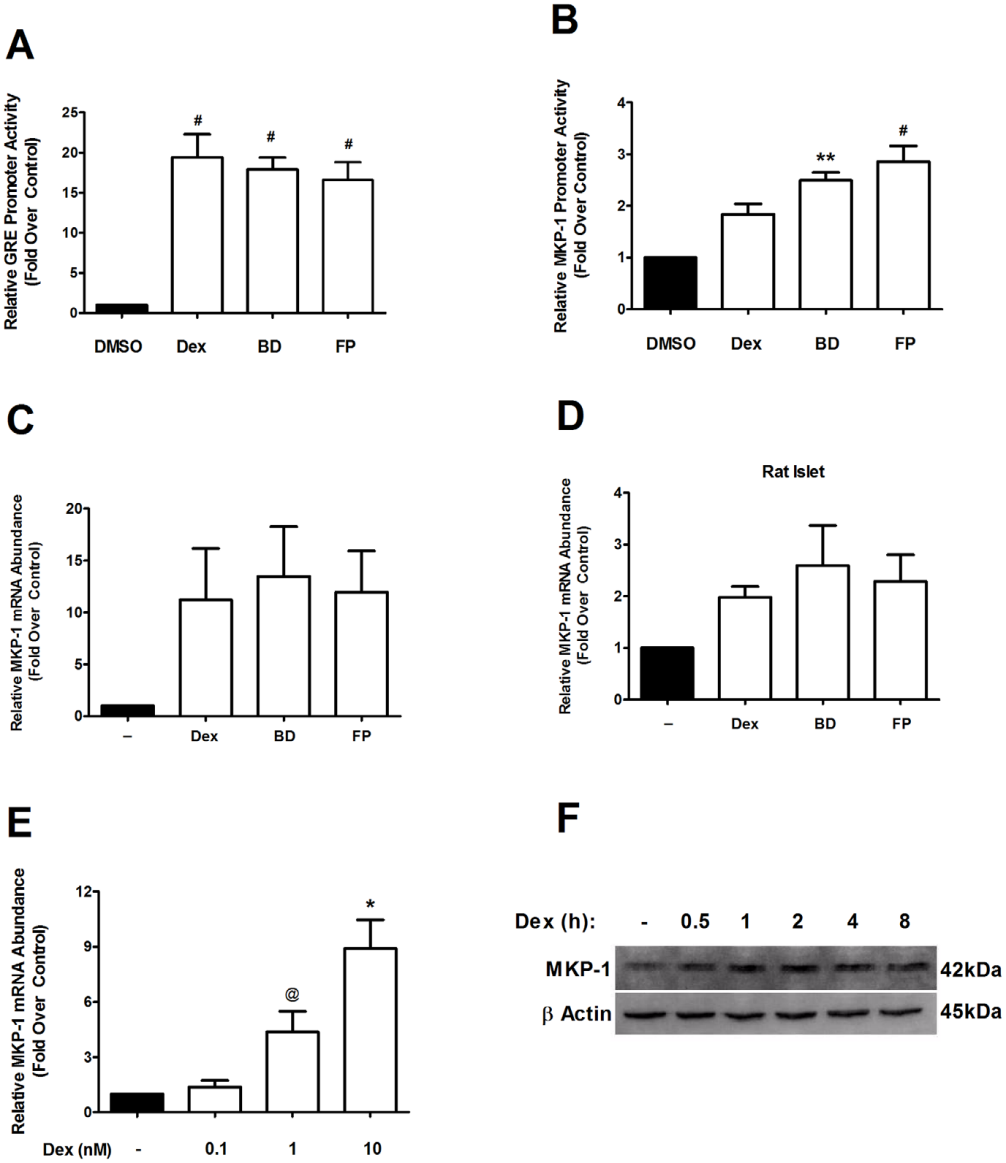
Activation of the glucocorticoid receptor increases expression of the MKP-1/DUSP1 gene and decreases IL-1β-stimulated p38 MAPK phosphorylation. *A, B*. 832/13 cells were transfected with either 3X GRE-luciferase (A) or MKP-1- luciferase (B); 24 h post-transfection cells were treated for 4 h with 10 nM Dex, BD or FP. Luciferase promoter activity was quantified and normalized to total cellular protein. ^#^ *p*<0.01 vs. DMSO (black bar), **p<0.01 vs. DMSO. *C*. 832/13 cells or *D*. Rat islets were treated with 10 nM Dex, BD or FP for 6 h. *E*. 832/13 cells were stimulated for 6 h with 0, 0.1, 1 or 10 nM Dex. *F*. 832/13 cells were treated with 10 nM Dex for 0, 3, 6 or 12 h. *B-F*. Total RNA was isolated and MKP-1 mRNA abundance was measured and normalized to RS9. ^@^ *p*<0.1 vs. DMSO, *p<0.05 vs. DMSO, n.s. = not significant vs. DMSO. *G*. 832/13 cells were treated with 10 nM Dex for 0.5, 1, 2, 4 or 8 h. Immunoblot analysis was performed with antibodies against MKP-1 using β Actin as a control for equal protein loading. The image shown is representative of two independent experiments. Data showing promoter activity and mRNA abundance are represented as means ± SEM from 3–4 individual experiments.

GCs promote expression of the MKP-1 gene [29], [31] and MKP-1 dephosphorylates the p38 MAPK [54]. Because GCs do not impair IL-1β-mediated translocation of p65 from cytoplasm to nucleus (Figure S5), and because expression of the CCL2 gene is p38 MAPK dependent (Figure 5), we next investigated whether the MKP-1 gene was responsive to GCs in 832/13 cells and isolated rat islets. First, we examined the effects of each glucocorticoid on a synthetic glucocorticoid response element (GRE) promoter luciferase construct. As shown in Figure 7A, Dex, BD, and FP induced GRE-luciferase promoter activity by 19.4, 17.9 and 16.6-fold respectively. Next, we used approximately 700 bases of the human MKP-1 proximal gene promoter, which contains multiple glucocorticoid responsive elements [34], and discovered that these sites supported a 1.8, 2.5 and 2.9-fold increase in transcriptional activity by the respective glucocorticoids (Figure 7B). We then examined expression of the endogenous MKP-1 gene and discovered that Dex, BD and FP increased MKP-1 mRNA 11.2-fold, 13.5-fold, and 12-fold, respectively in 832/13 cells (Figure 7C). The MKP-1 gene was also induced by the panel of glucocorticoids in isolated rat islets; although to a lesser extent than 832/13 cells (Figure 7D). Further examination of the MKP-1 gene in response to Dex revealed dose-dependent increases in MKP-1 mRNA accumulation (Figures 7E). We also observed a rapid increase in MKP-1 mRNA (not shown) and protein abundance in response to 10 nM dex (Figure 7F). The increase in MKP-1 protein does not directly correlate with the magnitude of induction seen for the mRNA encoding this protein. We interpret these data to indicate that signals downstream of transcription may also be involved in controlling accumulation of MKP-1 protein. Transcriptional and post-transcriptional regulation are not uncommon for genes involved in regulating sensitive cellular functions [55].Taken together, these observations indicate that GCs induce expression of the MKP-1 gene in pancreatic β-cells.

### Overexpression of MKP-1/DUSP1 Diminishes the IL-1β-mediated Increase in CCL2 Gene Expression and Secretion with Full Retention of β-cell Function

**Figure 8:**
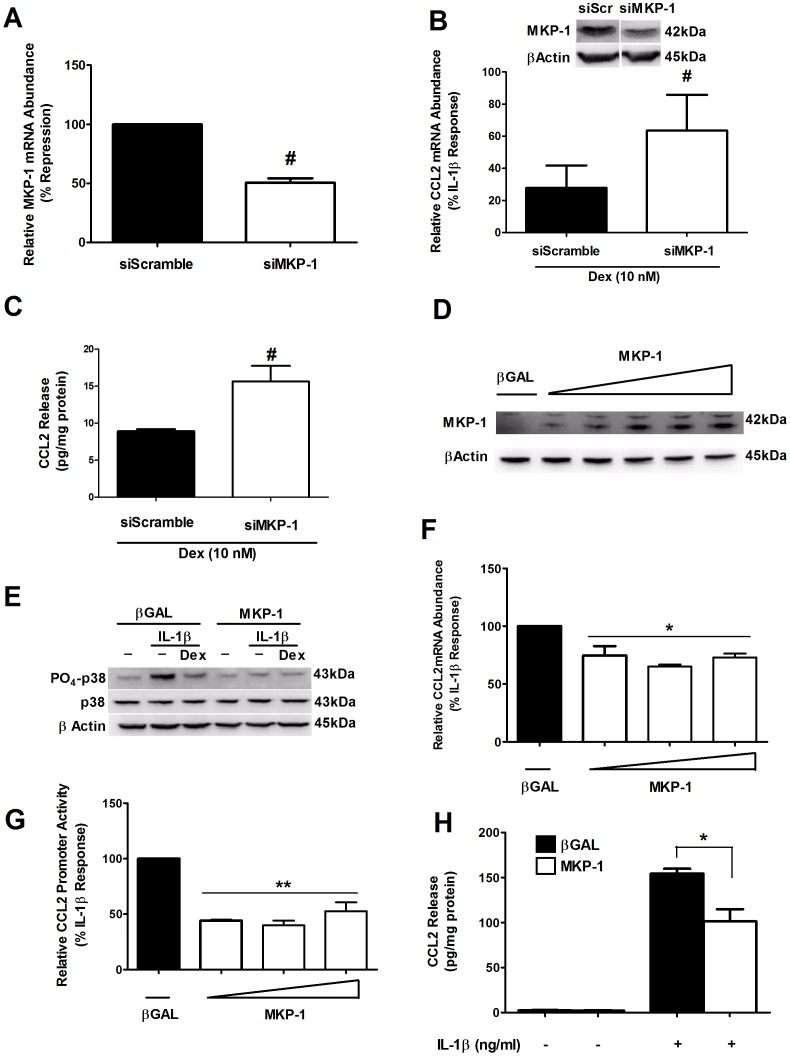
Overexpression of MKP-1 decreases the IL-1β-mediated activation of the CCL2 gene. *A–*C. 832/13 cells were transfected with siRNA duplexes targeting either a negative control sequence (siScramble) or siMKP-1. 24 h post-transfection cells were harvested and MKP-1 mRNA abundance was quantified (A), MKP-1 protein abundance was analyzed via immunoblot (B *inset*) or further treated concurrently with 10 nM Dex and 1 ng/mL IL-1β, then analyzed for CCL2 mRNA abundance (B) or CCL2 release into the media was measured via ELISA (C). **^#^** *p*<0.01 vs. siScramble. *D*. 832/13 cells were transduced with recombinant adenoviruses encoding either βGAL or MKP-1 (at multiple doses) overnight. Immunoblot analysis was done using whole cell lysates. The image shown represents two independent experiments. *E*. 832/13 cells were treated overnight with either βGAL or the lowest concentration of MKP-1 shown in *D*. The next day these cells were pre-treated with either DMSO (-) or 10 nM Dex for 6 h, followed by a 15 min exposure to 1 ng/mL IL-1β. Whole cell lysates were separated via SDS-PAGE. *F*. 832/13 cells were treated with either βGAL or three increasing concentrations of MKP-1; 24 h post-transduction cells were stimulated for 6 h with IL-1β. CCL2 mRNA abundance was measured and normalized to RS9. *p<0.05 vs. βGAL. *G*. 832/13 cells were treated with adenoviruses as in (F) for 4 h, transfected with 3.6-Luc and stimulated with IL-1β for 4 h. Promoter activity was normalized to total cellular protein. **p<0.01 vs. βGAL. *H*. 832/13 cells were transduced with either βGAL or MKP-1 adenovirus for 12 h and then incubated with IL-1β for a further 12 h. CCL2 release into the media was quantified via ELISA and data were normalized to total protein. *p<0.05. Data showing promoter activity, mRNA abundance and ELISA data are represented as means ± SEM from 3–4 individual experiments; promoter activity experiments were performed in duplicate or triplicate.

Using siRNA duplexes targeting the MKP-1 gene, we observed a 51% decrease in MKP-1 mRNA levels (Figure 8A); this reduction in MKP-1 coincided with an enhancement in IL-1β-stimulated p38 MAPK phosphorylation signal (not shown). Further, the siRNA-directed reduction in MKP-1 protein (Figure 8B -*inset*) eliminated 56% of the glucocorticoid-mediated suppression of the CCL2 gene (Figure 8B - *graph*). Moreover, this interference with Dex-mediated suppression resulted in 43% more CCL2 secretion (Figure 8C), demonstrating that the effectiveness of Dex to prevent CCL2 expression and release from the cell is diminished when MKP-1 abundance decreases.

To examine whether MKP-1 induction *per se* is sufficient to mimic the effects of Dex, we overexpressed MKP-1 in 832/13 cells using recombinant adenovirus (Figure 8D). Treatment with either Dex or overexpression of MKP-1 prevented the IL-1β-stimulated increase in p38 MAPK phosphorylation (Figure 8E). The dephosphorylation of p38 correlated with a reduction in IL-1β-mediated accumulation of CCL2 mRNA (Figure 8F). There was not a dose-dependent decrease in CCL2 expression in response to increasing concentrations of the MKP-1 virus (the range of suppression was 25–35%). Even more striking was the decrease in CCL2 gene transcription (Figure 8G), which was also not dose-dependent and included a range of reduction of 48–61%. Importantly, the secretion of CCL2 in response to IL-1β was also decreased by 30% via overexpression of MKP-1 (Figure 8H). These results are consistent with the model that inhibiting signaling through the p38 MAPK decreases the ability of IL-1β to induce expression of the gene and subsequent secretion of the CCL2 protein.

**Figure 9:**
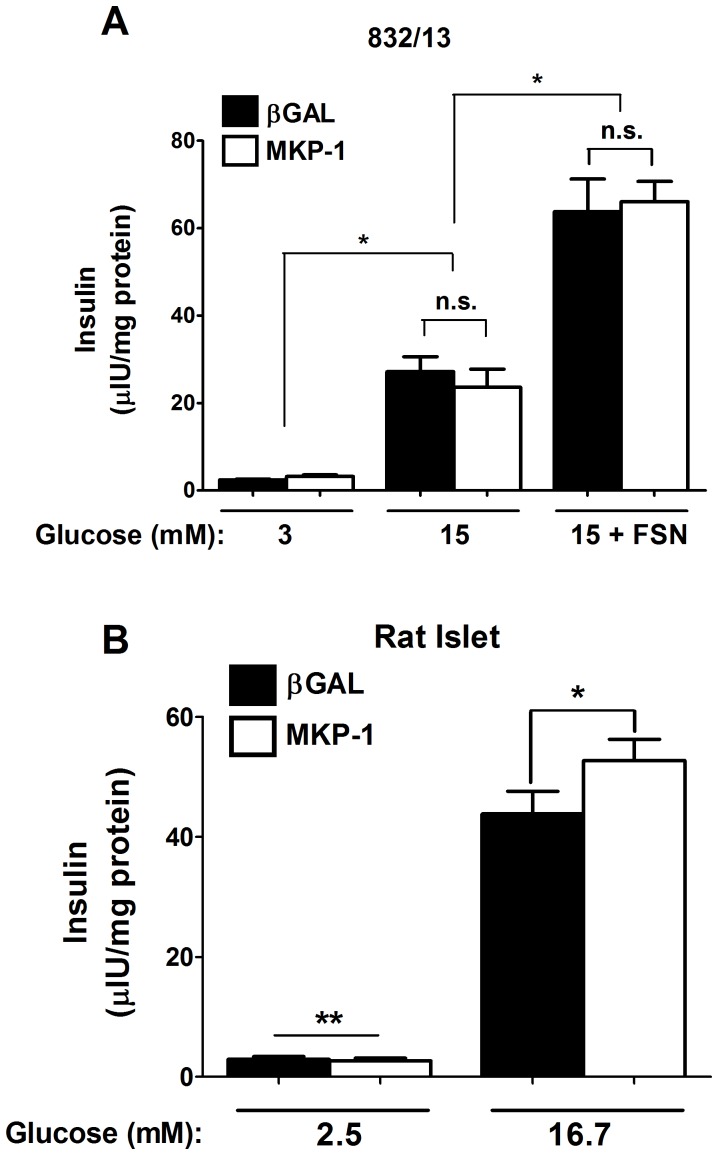
Overexpression of MKP-1 does not impair glucose-stimulated insulin secretion. *A*. 832/13 cells were transduced with the indicated recombinant adenoviruses overnight, followed by static incubation in either 3 mM or 15 mM glucose, or 15 mM glucose plus 5 µM forskolin. *B*. Isolated rat islets were transduced with the indicated adenoviruses for 48 h, followed by incubation in either 2.5 or 16.7 mM glucose. *A, B.* Insulin secreted into the culture media was measured by radioimmunoassay. *A*. *p<0.05 vs. respective glucose concentrations, n.s. = not significant; *B*. *p<0.05. All bar graphs shown represent means ± SEM from 3–4 individual experiments, some of which were performed in duplicate.

Of significant note, MKP-1 overexpression does not impair glucose-stimulated insulin secretion in 832/13 cells (Figure 9A). Moreover, the ability of forskolin (FSN), an adenylyl cyclase activator, to potentiate the stimulus-secretion coupling response by an additional 2.6-fold was also retained (Figure 9A). In addition, overexpression of MKP-1 in isolated rat islets also did not impair insulin secretion (Figure 9B). We observed that MKP-1 overexpression actually enhanced glucose-stimulated insulin secretion by 16.8% relative to the control virus expressing β-Galactosidase. Thus, an important finding here is that the anti-inflammatory actions associated with MKP-1 overexpression occur with full retention of β-cell function.

## Discussion

Chemokines play a crucial role in recruiting leukocytes to areas of inflammation [56], [57]. The chemokine CCL2 likely influences the islet immune cell recruitment related to T1DM, as well as with islet graft rejection [4], [8], [10], [18], [19], [58]. Thus, the present study was designed to examine the signals required to activate and repress expression of the CCL2 gene in pancreatic β-cells. Several novel observations emerged: 1) expression of the CCL2 gene in response to IL-1β requires the p65 subunit of NF-κB and signaling through the kinases IKKβ and p38 MAPK; 2) overexpression of p65 is sufficient to drive transcription of the CCL2 gene, leading to increased synthesis and secretion of CCL2; 3) multiple glucocorticoid receptor ligands diminish the IL-1β-mediated induction of the CCL2 gene; 4) overexpression of the glucocorticoid responsive gene MKP-1 partially impairs the ability of IL-1β to induce expression of the CCL2 gene and to promote CCL2 release; 5) overexpression of MKP-1 does not interfere with glucose-stimulated insulin secretion, demonstrating that the anti-inflammatory activity of this phosphatase occurs with preservation of β-cell secretory function.

The results reported herein are consistent with MKP-1 knockout animals which display exaggerated responses to innate immune receptor stimuli, including prolonged p38 phosphorylation [59]; this inability to dephosphorylate p38 in a normal manner is associated with an increase in circulating pro-inflammatory factors, including CCL2 [60]. In pancreatic β-cells, the exquisite sensitivity of the CCL2, but not the COX2 gene, to p38 inhibition is indicative of distinct signaling inputs required to facilitate efficient communication of the same signal (e.g., IL-1β). We note that while JNK is activated in pancreatic β-cells in response to IL-1β, this kinase does not appear to be modulated by MKP-1. However, the JNK pathway is required for repair of DNA damage after pro-inflammatory signals by upregulation of GADD45 expression in pancreatic β-cells [61] and thus represents the diverse actions associated with IL-1β signaling. Alternatively, the expression of the COX2 gene, which also requires p65 for induction by IL-1β [38], does not require p38 to communicate the IL-1β response (Figure 5C). We interpret these findings to indicate a tissue specific and/or signal specific phenomenon, as p38 is required to induce the expression of the COX2 gene in macrophages exposed to encephalomyocarditis virus [62]. We suspect that signal integration at various promoters is controlled by discrete mechanisms in order to finely tune homeostasis versus inflammation in a tissue specific manner.

The discovery that p65 overexpression is sufficient to drive synthesis and secretion of CCL2 from β-cells in the absence of a pro-inflammatory stimulus (Figure 3) has important implications for both major forms of diabetes. In our view, this observation indicates that any number of stimuli promoting degradation of the regulatory IκB proteins (e.g., cytokines, TLR-2 and -4 ligands, etc.) or otherwise facilitating p65 entry into the nucleus could potentially induce the expression of the CCL2 gene. In support of this view, fatty acids promote the synthesis and secretion of IL-1β through TLR-2 and -4 activation [63] leading to auto-inflammatory feed foward mechanisms that promote islet leukocyte accumulation [64]. CCL2 is one such gene that may be a contributor to this phenotype. Conversely, other IL-1β responsive genes, such as COX2, do not respond to simple increases in p65 abundance (not shown), indicating that the β-cell uses distinct and selective mechanisms to control the expression of genes associated with inflammation. The coupling of CCL2 gene transcription with CCL2 release allows the β-cell to rapidly respond to fluctuations in NF-κB activity with corresponding increases or decreases in chemoattractant potential.

Our observations are also congruent with several *in vivo* mouse studies in addition to findings related to islet transplantation. For example, transgenic overexpression of CCL2 in islet β-cells promotes massive immune cell infiltration into the islets [65] and the quantity of CCL2 synthesized and secreted from β-cells into the serum correlates with diabetes development [8]. Intriguingly, however, transgenic expression of CCL2 on the non-obese diabetic mouse background, a model of autoimmune-mediated islet destruction, delays diabetes development despite increased immune cell accumulation in islets [19]. Thus, genetic background potentially dictates the role of CCL2 involvement in diabetes onset and progression by potentially controlling the type of leukocyte recruited and the immune response pattern initiated (e.g., Th1 vs. Th2 responses).

In this study, we observed both synthesis and secretion of CCL2 in response to the pro-inflammatory cytokine IL-1β (Figure 1) a cytokine strongly implicated in the development of T1DM [66]. We further discovered that inhibition of the p38 MAPK strongly impaired both the synthesis and secretion of CCL2 (see Figures 5, 7 & 8). Our results may thus explain data from a previous report demonstrating that administration of a p38 inhibitor to NOD mice decreases islet immune cell infiltration, which delayed the development of overt diabetes [28]. Taken together, the data reported herein reveals a heretofore undescribed potential explanation for the decrease in insulitis during p38 inhibitor delivery to NOD mice *in vivo* (i.e., reduction in secreted CCL2 decreases insulitis). Additionally, we also established that discrete synthetic glucocorticoids prevented the IL-1β-stimulated increase in expression of the CCL2 gene (see Figure 6). These novel findings may also have relevance to autoimmunity in the NOD mouse since these animals exhibit decreases in glucocorticoid receptor abundance prior to onset of diabetes [67]. The decline in GR protein may render the animals less sensitive to circulating glucocorticoids and therefore predispose the mice towards autoimmune and autoinflammatory conditions, including the development of T1DM.

Treatment of T1DM by islet transplantation is still a work in progress [68], [69]. Strategies to improve islet function and prevent graft rejection are critical barriers to successful transplantation therapy. Acute treatment of human islets *ex vivo* with glucocorticoids *prior to* transplantation improved their function *after* transplantation [70]. This improvement in function could be due in large part to suppression of soluble secreted factors (e.g., CCL2, IL-8, etc.) that stimulate the recruitment of immune cells into grafted tissue. In addition, GC-mediated increases in anti-inflammatory proteins, such as MKP-1 (discussed below), are likely to also play key roles. While chronic glucocorticoid usage is associated with multiple negative side effects and was removed from some islet transplantation protocols, we propose that shorter term exposures may be useful for opposing the powerful pro-inflammatory signals that impair β-cell function and viability. Furthermore, modest doses of dexamethasone injected into rats enhanced glucose-stimulated insulin secretion in the subsequently isolated islets [71]. Thus, it is plausible that an enhancement in islet function may initially be possible in acute glucocorticoid treated islets, but long-term, chronic activation of the glucocorticoid receptor systemically could be detrimental to both pancreatic islets as well as peripheral tissues. Consequently, alternative strategies to suppress inflammation with the goal of retaining long-term islet function would circumvent the harmful side effects of chronic glucocorticoid exposure. It is therefore plausible that manipulation of GC responsive genes may be one such strategy.

**Figure 10:**
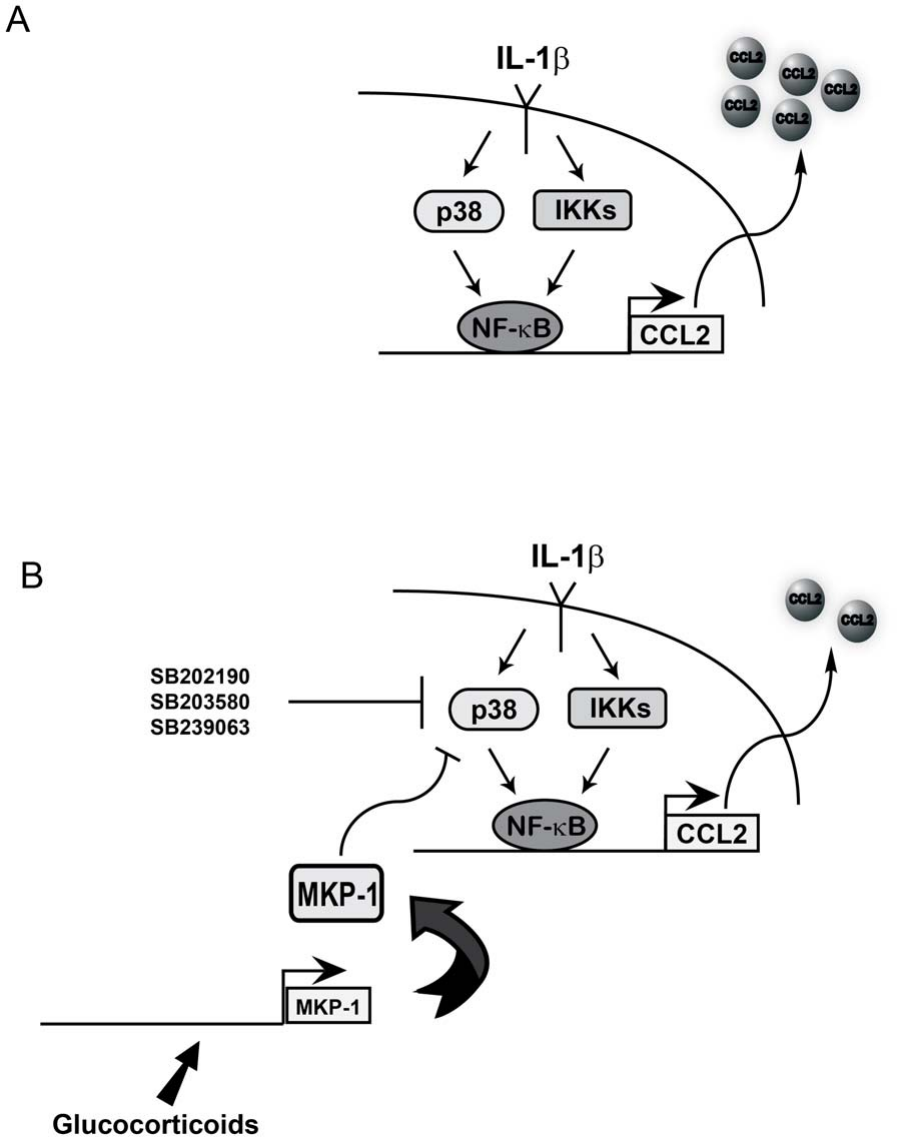
Pharmacological or phosphatase-mediated inhibition of IL-1β-stimulated p38 MAPK phosphorylation decreases synthesis and secretion of CCL2. *A.* IL-1β induces release of NF-κB subunits from regulatory proteins by activating the IKKs. In addition, IL-1β promotes phosphorylation of p38 MAPK, increases CCL2 transcription using the NF-κB pathway, culminating with secretion of CCL2 protein. *B.* The activation of p38 by IL-1β is critical for induction of CCL2 gene expression and secretion of CCL2 protein. Inhibiting p38 MAPK activity, either through the use of pyridinyl-imidazole based inhibitors or via MKP-1 mediated dephosphorylation, impairs synthesis and secretion of CCL2.

Towards this end, we examined the GR controlled gene MKP-1 (aka DUSP-1) as a potential regulatory factor controlling IL-1β-mediated signaling in the pancreatic β-cell. We selected MKP-1 because it has been well studied as both a gene induced by GCs [29], [34] and because the knockout mice displayed enhanced sensitivity to inflammatory stimuli [59], [60]. Since both glucocorticoids and p38 inhibitors were effective strategies to oppose the effects of IL-1β, it was reasonable to postulate that MKP-1 might be an effective and targeted anti-inflammatory strategy. We demonstrate here for the first time that multiple GR ligands induced the expression of the MKP-1 gene in isolated rat islets and β-cell lines, leading to a decrease in IL-1β-stimulated p38 MAPK phosphorylation (Figure 10). Interfering with GR-mediated induction of MKP-1 by siRNA duplexes decreased the effectiveness of GCs to suppress CCL2 gene expression (Figure 8). We interpret this finding to indicate that upregulation of this phosphatase by GCs is a key component to controlling signal intensity to the CCL2 gene, and perhaps other genes. Moreover, the effects of MKP-1 overexpression to oppose p38 signaling also suppressed CCL2 release (Figure 8). We note that MKP-1 overexpression had a more modest impact on the CCL2 gene when compared to pharmacological inhibitors of p38 MAPK or to the effects of glucocorticoids. We attribute this finding to the pharmacological inhibitors being able to suppress both stimulated *and* tonic activity of the p38 MAPK (by virtue of competing with ATP for active site binding), while the phosphatase can only diminish stimulus-enhanced activity by removing phosphate groups from specific amino acids. On the other hand, GCs can potentially employ a variety of mechanisms to suppress inflammation, of which inducing MKP-1 is only one such possibility. Indeed, silencing MKP-1 only partially inhibited the effectiveness of GCs to suppress CCL2 expression and secretion, while overexpression of MKP-1 did not fully mimic the potency of GCs. These data are consistent with another recent report using a different cellular population [72].

Moreover, it is likely that glucocorticoids employ multiple mechanisms in order to suppress inflammatory responses and induction of phosphatase proteins, such as MKP-1, represent only one important component of GR action. It is possible, even likely, that other phosphatases of the dual specificity family may be coordinately regulated by glucocorticoids to tailor inflammatory responses to the individual needs of a particular cell. Furthermore, ligand-bound GR may directly interfere with transcriptional machinery at various promoters, as has been described in other systems [73]. Thus, MKP-1 overexpression would perhaps be predicted to only partially recapitulate the actions associated with liganded GR, a postulate consistent with what we observed in the present study. It thus remains to be determined how many of the DUSP family members are responsive to GCs in the pancreatic β-cell and if any of these other phosphatases represent anti-inflammatory actions that could be exploited to protect β-cells from pro-inflammatory signals. Moreover, a direct repressive effect of liganded GR on specific genes activated by IL-1β cannot be ruled out at the present time. It is entirely possible, even likely, that GCs use multiple mechanisms to suppress inflammation in pancreatic β-cells, while MKP-1 simply targets a highly specific pathway (e.g., MAPKs).

Finally, the preservation of β-cell secretory capacity is a significant consideration for any anti-inflammatory strategy aimed at maintaining or restoring functional β-cell mass. Notably, neither glucose-stimulated insulin secretion nor the potentiating effects of cAMP on glucose-stimulated insulin secretion were impaired with MKP-1 overexpression. Elevated cAMP levels are essential for the actions of incretin hormones on pancreatic β-cell function. The insulin secretion results obtained after delivering the MKP-1 gene to rat islets and β-cell lines are fundamentally important considering that decreases in β-cell function are a known side effect of chronic glucocorticoid exposure [74], [75]. We have shown here that MKP-1 gene delivery modestly enhances islet β-cell function, which is consistent with studies that have measured insulin secretion in isolated islets in the presence of p38 MAPK inhibitors [26], [76]. Finally, we note that annexin A1, itself a glucocorticoid-responsive anti-inflammatory gene in some tissues [77], is not responsive to dexamethasone in the pancreatic β-cell (JJC, unpublished data). Therefore, individual tissues clearly use distinct strategies to combat inflammation; accordingly, glucocorticoids induce and/or repress discrete and overlapping genes in different tissues towards achieving this goal.

In summary, the separation of the anti-inflammatory properties of glucocorticoids from their side effects is an ongoing pursuit of many laboratories; consequently, glucocorticoid responsive genes might represent a novel approach for therapeutic intervention in many tissues. While we have described the role of MKP-1 in the current study, future studies will include additional dissection of glucocorticoid receptor-mediated actions, including analysis of other downstream target genes, in an attempt to identify additional strategies which can protect pancreatic β-cells from pro-inflammatory signals. Thus, we conclude that increased expression of the MKP-1 gene, either via GC exposure or via viral overexpression, is a negative regulator of IL-1β-mediated p38 activation. Interfering with p38 activity by pharmacologic or gene delivery approaches reduces the expression of the CCL2 gene in response to IL-1β, which results in diminished levels of secreted CCL2 protein. Importantly, these actions were achieved with full retention of β-cell function.

## Supporting Information

Figure S1 **Cycle threshold (Ct) values reflecting relative expression patterns of the CCL2, Pdx1, Nkx6.1 and GADD45 genes in 832/13 rat insulinoma cells either at basal levels or stimulated with 1 ng/ml IL-1β for 6 hrs.** Numerical values are *average* Ct values, representing three independent RT-PCR runs.
(TIF)

Figure S2 **832/13 cells were transfected with either an siRNA duplex targeting p65 or non-targeting siScramble control.** Following 48 h incubation with duplexes cells were harvested and separated into cytoplasmic and nuclear fractions. Shown is a representative immunoblot of p65 protein abundance, with β-Actin serving as a loading control.
(TIF)

Figure S3 **832/13 cells were pre-treated for 1 hr with 1 µM SP600125 (JNKi) followed by a 6 h stimulation with 1 ng/ml IL-1β alone or in combination with 1 U/ml γ-IFN for 6 hrs.** GADD45 mRNA abundance was measured and normalized to RS9. Data are means ± SEM from 3 individual experiments. *p<0.05.
(TIF)

Figure S4 **832/13 cells were transfected with the 3X GAS- luciferase construct; 24 h post-transfection cells were treated for 1 h with 20 µM ERK inhibitor prior to 4 h stimulation with 100 U/mL γ-IFN.** Luciferase reporter activity was normalized to protein content. Data are represented as means ± SEM from 3 individual experiments. *p<0.05.
(TIF)

Figure S5 **832/13 cells were treated with 10 nM Dex for 1 h then stimulated with 1 ng/ml IL-1β for 15 mins.** Immunofluorescence assay was used to track nuclear localization of p65 (Scale bars represent 50 µm on the main image and 5 µm scale bars within the magnified inlays). Immunofluorescence experiments were conducted on three individual occasions and representative images are shown.
(TIF)
