# Supplementary figures and images for: Regulation of the CCL2 Gene in Pancreatic β-Cells by IL-1β and Glucocorticoids: Role of MKP-1
Source: PLoS One. 2012 Oct 9;7(10):e46986. doi: 10.1371/journal.pone.0046986 (PMC3467264; doi:10.1371/journal.pone.0046986)

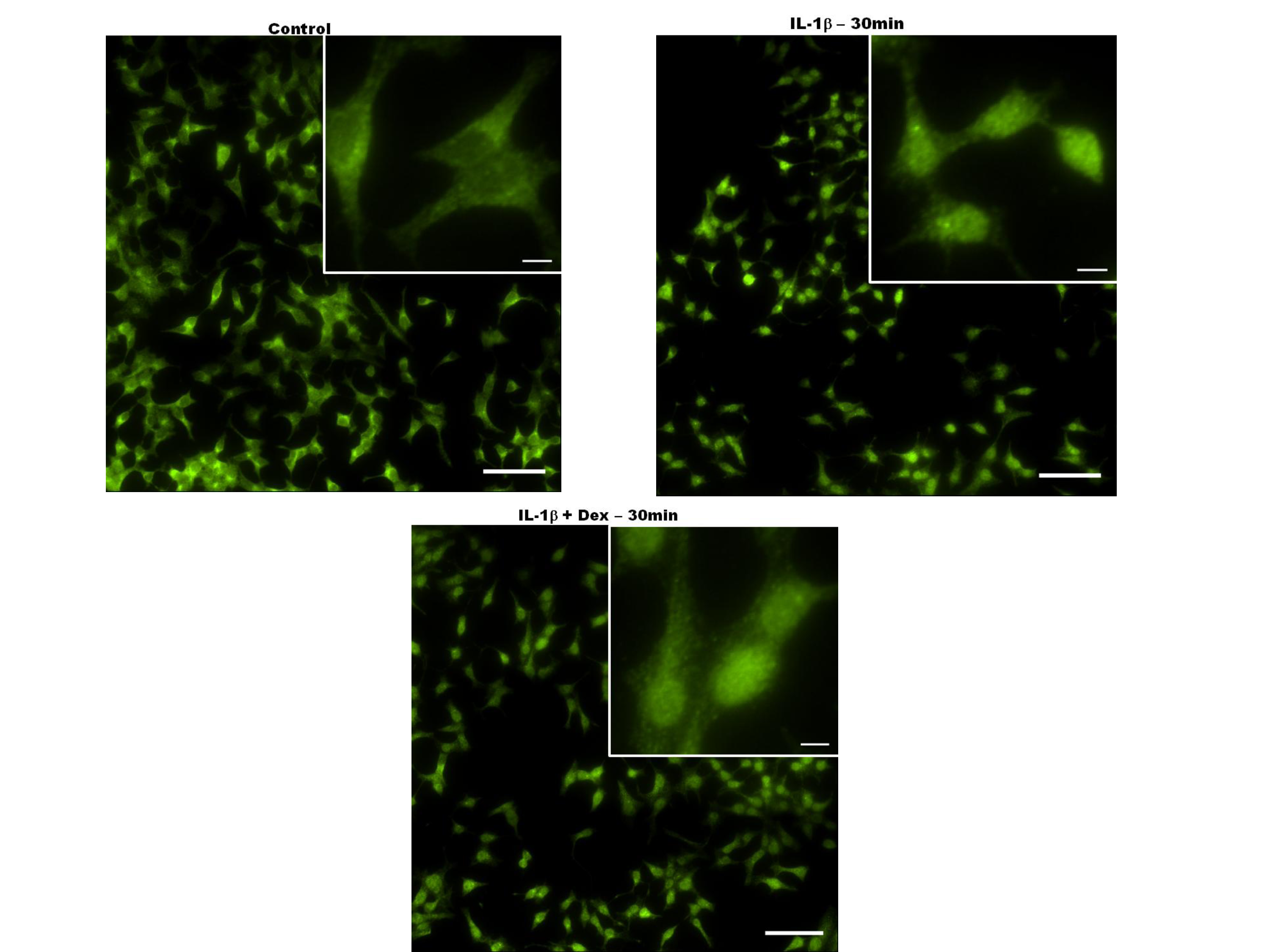

Supplement: Figure S5 — 832/13 cells were treated with 10 nM Dex for 1 h then stimulated with 1 ng/ml IL-1β for 15 mins. Immunofluorescence assay was used to track nuclear localization of p65 (Scale bars represent 50 µm on the main image and 5 µm scale bars within the magnified inlays). Immunofluorescence experiments were conducted on three individual occasions and representative images are shown. (TIF) [file pone.0046986.s005.tif]
